# Characteristics and Admission Preferences of Pediatric Emergency Patients and Their Waiting Time Prediction Using Electronic Medical Record Data: Retrospective Comparative Analysis

**DOI:** 10.2196/49605

**Published:** 2023-11-01

**Authors:** Lin Lin Guo, Lin Ying Guo, Jiao Li, Yao Wen Gu, Jia Yang Wang, Ying Cui, Qing Qian, Ting Chen, Rui Jiang, Si Zheng

**Affiliations:** 1 Children's Hospital Capital Institute of Pediatrics Beijing China; 2 Institute of Medical Information Chinese Academy of Medical Sciences and Peking Union Medical College Beijing China; 3 Department of Computer Science and Technology Tsinghua University Beijing China; 4 Department of Automation Tsinghua University Beijing China

**Keywords:** pediatric emergency department, characteristics, admission preferences, waiting time, machine learning, electronic medical record

## Abstract

**Background:**

The growing number of patients visiting pediatric emergency departments could have a detrimental impact on the care provided to children who are triaged as needing urgent attention. Therefore, it has become essential to continuously monitor and analyze the admissions and waiting times of pediatric emergency patients. Despite the significant challenge posed by the shortage of pediatric medical resources in China’s health care system, there have been few large-scale studies conducted to analyze visits to the pediatric emergency room.

**Objective:**

This study seeks to examine the characteristics and admission patterns of patients in the pediatric emergency department using electronic medical record (EMR) data. Additionally, it aims to develop and assess machine learning models for predicting waiting times for pediatric emergency department visits.

**Methods:**

This retrospective analysis involved patients who were admitted to the emergency department of Children’s Hospital Capital Institute of Pediatrics from January 1, 2021, to December 31, 2021. Clinical data from these admissions were extracted from the electronic medical records, encompassing various variables of interest such as patient demographics, clinical diagnoses, and time stamps of clinical visits. These indicators were collected and compared. Furthermore, we developed and evaluated several computational models for predicting waiting times.

**Results:**

In total, 183,024 eligible admissions from 127,368 pediatric patients were included. During the 12-month study period, pediatric emergency department visits were most frequent among children aged less than 5 years, accounting for 71.26% (130,423/183,024) of the total visits. Additionally, there was a higher proportion of male patients (104,147/183,024, 56.90%) compared with female patients (78,877/183,024, 43.10%). Fever (50,715/183,024, 27.71%), respiratory infection (43,269/183,024, 23.64%), celialgia (9560/183,024, 5.22%), and emesis (6898/183,024, 3.77%) were the leading causes of pediatric emergency room visits. The average daily number of admissions was 501.44, and 18.76% (34,339/183,204) of pediatric emergency department visits resulted in discharge without a prescription or further tests. The median waiting time from registration to seeing a doctor was 27.53 minutes. Prolonged waiting times were observed from April to July, coinciding with an increased number of arrivals, primarily for respiratory diseases. In terms of waiting time prediction, machine learning models, specifically random forest, LightGBM, and XGBoost, outperformed regression methods. On average, these models reduced the root-mean-square error by approximately 17.73% (8.951/50.481) and increased the R^2^ by approximately 29.33% (0.154/0.525). The SHAP method analysis highlighted that the features “wait.green” and “department” had the most significant influence on waiting times.

**Conclusions:**

This study offers a contemporary exploration of pediatric emergency room visits, revealing significant variations in admission rates across different periods and uncovering certain admission patterns. The machine learning models, particularly ensemble methods, delivered more dependable waiting time predictions. Patient volume awaiting consultation or treatment and the triage status emerged as crucial factors contributing to prolonged waiting times. Therefore, strategies such as patient diversion to alleviate congestion in emergency departments and optimizing triage systems to reduce average waiting times remain effective approaches to enhance the quality of pediatric health care services in China.

## Introduction

The main objective of China’s health care reform was to improve health care quality and increase public satisfaction with all health care services [1,2]. Currently, relevant policies and a series of actions are being implemented by different hospitals, yet more progress is clearly needed, as many problems remain to be solved [3,4]. For instance, similar to previous studies, increased visit volume may result in overcrowding in the emergency department (ED) and increased waiting times for minor and sometimes serious problems [5,6]. Defined by the Food and Drug Administration as data related to a patient’s health status, real-world data are increasingly being used in clinical decision-making [7]. Analyzing real-world data, such as electronic medical records, allows researchers to discover critical factors that may not be visible in smaller sample sizes and to evaluate potential collaborations in building efficient task-sharing models of health care [8-11].

Pediatric emergency care poses distinctive safety challenges compared with regular general practice. Physicians in this setting must make rapid and precise assessments despite limited time and resources, given the vulnerability of the patient population. The increasing number of patients presenting to pediatric EDs (PEDs) can have adverse effects on the care provided to acutely ill and injured children. This may result in diminished health care quality, negative clinical outcomes, and reduced patient satisfaction [12-14]. As a result, PEDs constantly grapple with the task of aligning their resources with the increasing demand for emergency care. Past research has indicated various measures aimed at mitigating these issues. These include efforts to reduce ED attendance through the reconfiguration and promotion of community services, as well as the implementation of triage systems and early warning scores to prioritize all children in PEDs [15,16]. Furthermore, there have been advances in the development of tools for measuring PED crowding, such as PEDOCS (Pediatric ED Overcrowding Score) [17] and SOTU-PED (a real-time crowding composite scale for pediatric emergency department) [18]. However, their reliability and effectiveness in various ED settings remain uncertain. Additionally, many of the current ED crowding assessment tools were created by expert panels, and some of the factors they consider are meant to reflect crowding in adult EDs rather than in pediatric ones. This may make them less applicable to pediatric environments [19]. Consequently, for the practical allocation of medical resources, it is necessary to continuously analyze the real-world data of pediatric emergency attendances; summarize their demographic characteristics, clinical presentation, and medical visit patterns; and infer the likely causes and patterns of the increasing number of arrivals as well as the prolonged waiting times.

Waiting time to access consultation or treatment is an important component of the quality of the overall health care experience. Previous studies have applied regression models or rolling average estimations to predict waiting time [20,21]. For instance, Ataman et al [22] proposed an ordinal logistic regression model to predict waiting times in EDs and identified age, arrival mode, and ICD-10 (International Classification of Diseases 10th Revision)–encoded diagnoses as significant predictors. Eiset et al [23] built a transition regression model to estimate departures from the ED, which can be used to predict the expected waiting time and crowding in the ED. However, these methods have limited accuracy, as they do not account for the dynamic and complex nature of the delivery of ED services. Recently, machine learning models have been widely used to improve prediction accuracy. Pak et al [24] used machine learning algorithms to generate more accurate ED waiting time predictions than regression models. Machine learning algorithms using systems knowledge could significantly improve the performance of waiting time prediction and offer considerable advantages for health care improvement [25,26]. However, most previous studies primarily aimed at predicting waiting times in general ED populations, with only a limited focus on pediatric emergency patients. In practice, the scarcity of pediatric medical resources presents a significant challenge to China’s health care system. There has been relatively little research that continuously analyzes extensive visits to PEDs in Chinese children’s hospitals. This type of analysis is critical for optimizing medical resource allocation and enhancing the quality of pediatric health care.

In response to these challenges, we conducted a comprehensive retrospective study to assess the pediatric patient profile in the ED of a children’s hospital in northern China. Our goal was to analyze their admission preferences, offer real-time and precise waiting time estimates and predictions for the PED, and formulate policy recommendations for both medical resource allocation and pediatric emergency management.

## Methods

### Study Design

**Figure 1:**
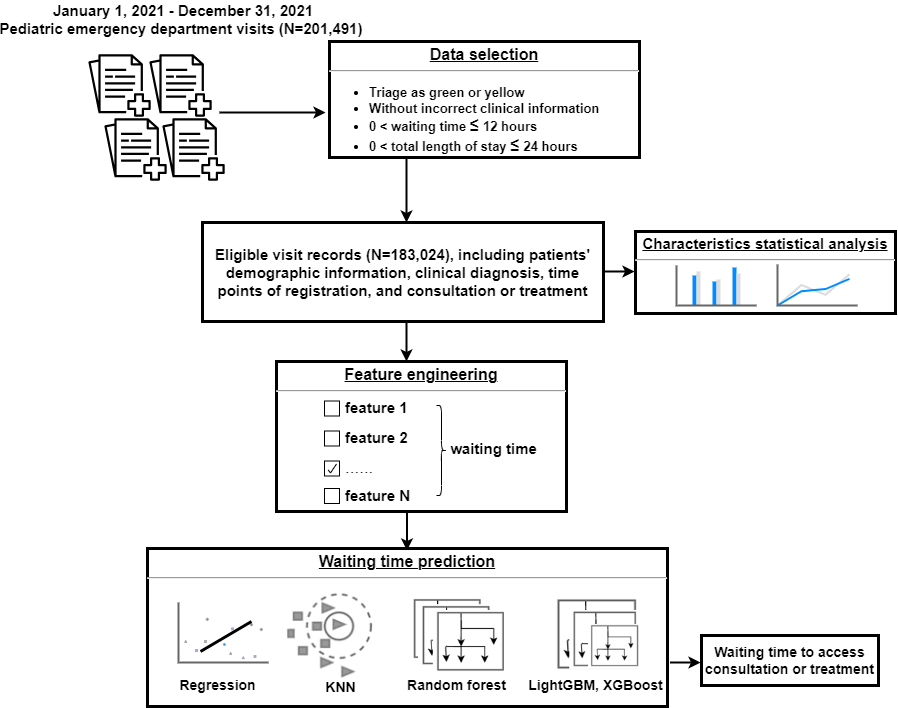
Workflow for analyzing the characteristics and waiting time of pediatric emergency department visits. KNN: K-nearest neighbor; LightGBM: light gradient-boosting machine; XGBoost: extreme gradient boosting.

In this retrospective study, we analyzed deidentified clinical data from pediatric patients who were admitted to the Children’s Hospital Capital Institute of Pediatrics’ ED between January 1, 2021, and December 31, 2021. Our study aimed to evaluate the demographic characteristics, clinical presentations, and medical visit patterns of these PED visits. We used various computational models for predicting waiting times and discussed factors associated with increased arrivals and extended waiting times (Figure 1).

### Data Collection

We identified all patients admitted to the Children’s Hospital Capital Institute of Pediatrics’ ED between January 1, 2021, and December 31, 2021, using the hospital’s information system. There were a total of 201,491 PED visits from 136,225 anonymous patients over approximately 12 months. We gathered comprehensive medical information about each patient’s PED journey. Essentially, we recorded key time points for each PED visit, including registration, doctor consultation or treatment, prescription filling, and departure. Upon arrival and registration, triage nurses assessed the patients’ clinical conditions, and the triaged patients then waited to see a physician. Throughout their hospital journey, some patients receive their medication and leave without revisiting, while others require additional tests for their initial diagnosis. These patients return to the waiting room for further clinical consultation or treatment after these tests. In this study, the waiting time to see a doctor was calculated from the moment of registration upon arrival to the first physician visit. Additional collected data encompassed patient gender, age, registration department, triage levels, diagnosis, and the number of doctors on duty.

### Data Preprocessing and Data Analysis

#### Data Selection and Variable Definitions

We excluded the following PED visit records: (1) those with incorrect clinical records, such as patients admitted when they were over 18 years of age; (2) those with unusually short or long waiting times (below 0 or above 12 hours, as the PED registration is valid for only up to 12 hours in this hospital); and (3) those with exceptionally long total lengths of stay (over 24 hours). Furthermore, we excluded patients who were clinically triaged as red or those without triage information. We made this exclusion based on the assumption that patients with red tags constituted a relatively small subgroup and always received immediate treatment, with their medical resources not shared with others. In this study, we focused on patients triaged as “green” or “yellow,” as they represent the majority of PED visits and waiting queues, and typically have a lower priority for medical treatment. A total of 183,024 eligible records were retained.

For the waiting time prediction task, we selected 27 predictive variables based on inspiration from previous studies [24,25] (see Multimedia Appendix 1). These features comprehensively describe overcrowding in PEDs, encompassing patient status, fluctuations in current/previous PED activities, categories of the current time stamp, etc. The primary outcome for model prediction was the waiting time to access consultation or treatment.

#### Statistical Analysis

We performed statistical analysis to summarize and compare patient characteristics and waiting times. Because of the highly left-skewed distribution of waiting times, we used the median to describe central tendency. Median waiting times were compared among patient groups with different triage levels, registration departments, and age groups using the Wilcoxon rank sum test. A significance level of *P*<.01 was applied, and R version 4.2.2 (The R Foundation) was used for statistical analysis.

### Model Construction and Feature Importance Analysis

We used 8 models to predict waiting times for PED visits. The rolling average estimator and linear regression (LR) were used as baseline models, while machine learning methods, including K-nearest neighbor (KNN), random forest (RF), LightGBM (light gradient-boosting machine), and XGBoost (extreme gradient boosting), were also used. The rolling average calculates short-term trends using a set of data. In short, with *t_reg_* as the registration time stamp of a patient and *n* as a rolling time, the rolling average estimator predicts a patient’s waiting time by calculating the average waiting time of patients whose registration time stamps *t*′_reg_ and consultation or treatment time stamps *t*′_con_ fall within the interval *t*_reg_ – n, *t*_reg_. In our study, we set *n* = {4h,2h} and constructed 2 rolling average estimators called Rol.Avg.4 h and Rol.Avg.2 h.

We used 10-fold cross-validation to evaluate the model performance. Additional information on the model hyperparameter search experiments conducted during this process is available in Multimedia Appendix 2. To mitigate random errors resulting from data splitting, we performed 10 repeated cross-validations with different random seeds and reported the average performance and SD for linear regression and machine learning methods. Additionally, we used Shapley additive explanation values, a successful tool in clinical predictions [27,28], to evaluate variable importance and select those most influential in waiting time prediction.

In our study, we used a Python script (Python Software Foundation) that used various imported application programming interfaces (scikit-learn=0.23.2, xgboost=1.2.0, lightgbm=3.0.0, shap=0.39.0) for model development. Model performance was assessed using *R*^2^, mean absolute error, and root-mean-square error (RMSE).

### Ethics Approval

All methods were performed in accordance with the relevant guidelines and regulations. As we used anonymized and deidentified data and did not constitute human research, the need for written informed consent was waived by the Ethics Committee of Children’s Hospital Capital Institute of Pediatrics due to the retrospective nature of the study (approval number SHERLLM2022034).

## Results

### Characteristics of the Pediatric Emergency Department Visits

We collected data on a total of 183,024 eligible PED visits, involving 127,368 pediatric patients, during the study period. Of these, 56.90% (104,147/183,024) were male patients and 43.10% (78,877/183,024) were female patients (Table 1). The PED had an average of 501.44 visits/day (range 179-1145 visits/day). The majority of eligible admissions (175,791/183,024, 96.05%) were assigned to the green zone, indicating fewer complex conditions and less urgent treatment categories. Furthermore, we observed repeated visits to the PED, with 9.12% (11,612/127,368) of patients undergoing more than 2 emergency visits during the study period. Additionally, 18.76% (34,339/183,024) of PED visits resulted in discharge without a prescription or further tests.

The median waiting time from registration to seeing a doctor upon arrival was 27.53 minutes (IQR 8.48-83.25 minutes). Furthermore, 43.59% (79,786/183,024) waited less than 20 minutes, and 66.72% (122,120/183,024) waited less than 60 minutes. However, nearly one-fifth of patients waited over 100 minutes for clinical consultation or treatment. Comparatively, daytime PED visits had a median waiting time of 21.92 minutes, shorter than nighttime visits (35.90 minutes). Additionally, patients triaged in the yellow zone had a median waiting time of less than one-third of those triaged in the green zone (Table 1). Furthermore, pediatric patients registered in the emergency internal medicine department experienced longer waiting times compared with those in the emergency surgery department (median waiting time: 38.57 minutes versus 8.35 minutes, respectively; *P*<2.2×10^–16^). This may be because the emergency surgery department often handles relatively simple procedures, such as dressing changes or minor wound and burn treatments. Pediatric patients with moderate to severe symptoms typically received emergency care in the internal medicine department, which often resulted in longer waiting times.

**Figure 2:**
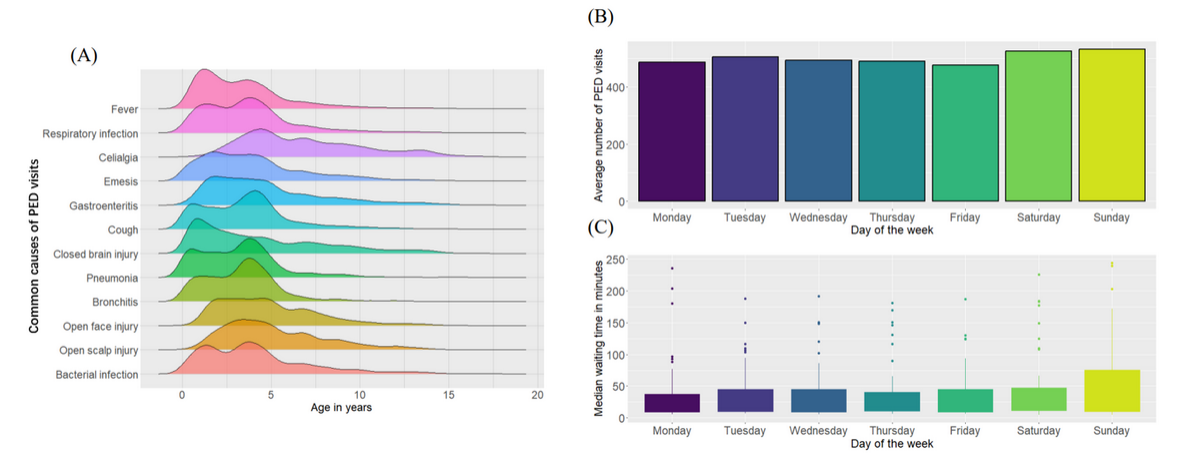
(A) Ridgeline plots between age and common diseases of PED visits. Density plot for each group is represented by different colors. (B) The average daily number of PED visits at different days of the week. (C) The box plot shows the daily median waiting time (minutes) at different days of the week. PED: pediatric emergency department.

In this retrospective study, 71.26% (130,423/183,024) of PED visits were from children under 5 years of age, and 41.65% (76,228/183,024) were from those under the age of 3 years. The mean age of PED visits was 4.14 (SD 3.18) years. Patients under the age of 3 years had shorter waiting times compared with other groups (*P*<2.2×10^–16^), with median waiting times of 25.80 and 28.82 minutes, respectively. Children with no more than 2 PED visits had a higher percentage of longer waits compared with those with 3 or more visits (median waiting time: 28.85 minutes versus 23.93 minutes). Fever (50,715/183,024, 27.71%), respiratory infection (43,269/183,024, 23.64%), celialgia (9560/183,024, 5.22%), and emesis (6898/183,024, 3.77%) were the leading causes of emergency room visits among children. Patients with respiratory tract infections often have fever symptoms. As depicted in Figure 2A, pediatric patients presenting with fever, respiratory infections, cough, pneumonia, bronchitis, or bacterial infections in the ED exhibited similar bimodal distributions across various age groups, specifically in the 1-2– and 3-4–year-old categories. When comparing the frequency of the top 20 leading causes of PED visits in 2 age groups (under 5 years old and 5 years old and older), several respiratory diseases (bronchitis, n=3227; pneumonia, n=2173; upper respiratory tract infection, n=1119; asthmatic bronchitis, n=1040; and laryngitis, n=926) were observed as common causes in the under 5-year-old age group, while more general symptoms (chest pain, n=626; headache, n=572; dizziness, n=432; and chest distress, n=411) occurred in the 5-year-old and older age group.

**Table 1:** Selected demographic characteristics and waiting time of PED^a^ visits between January and December 2021.

Characteristics			Values (N=183,024)
**Total number of PED visits**			
	**Triage zone, n (%)**		
		Yellow	7233 (3.95)
		Green	175,791 (96.05)
	**Department, n (%)**		
		Emergency surgery	28,595 (15.62)
		Emergency internal medicine	154,429 (84.38)
	**Gender, n (%)**		
		Male	104,147 (56.90)
		Female	78,877 (43.10)
	Age (years), mean (SD)		4.14 (3.18)
**Number of visits in queue upon arrival**			
	**Number of visits in queue for first consultation or treatment, mean (SD)**		
		Daytime (8:00 AM-8:00 PM)	25.28 (26.20)
		Nighttime (8:00 PM-8:00 AM)	31.29 (27.96)
	**Number of visits in queue for further consultation or treatment, mean (SD)**		
		Daytime (8:00 AM-8:00 PM)	21.11 (9.69)
		Nighttime (8:00 PM-8:00 AM)	20.24 (10.45)
	**Number of doctors on duty, mean (SD)**		
		Daytime (8:00 AM-8:00 PM)	13.62 (2.99)
		Nighttime (8:00 PM-8:00 AM)	12.04 (2.94)
**Waiting time (minutes)**			
	**Department, median (SD)**		
		Emergency surgery	8.35 (18.44)
		Emergency internal medicine	38.57 (76.60)
	**Triage zone, median (SD)**		
		Yellow	7.67 (56.97)
		Green	29.73 (73.47)
	**Gender, median (SD)**		
		Male	25.83 (72.60)
		Female	29.87 (74.13)

^a^PED: pediatric emergency department.

### Pediatric Emergency Department Admission Trends and Their Waiting Time

**Figure 3:**
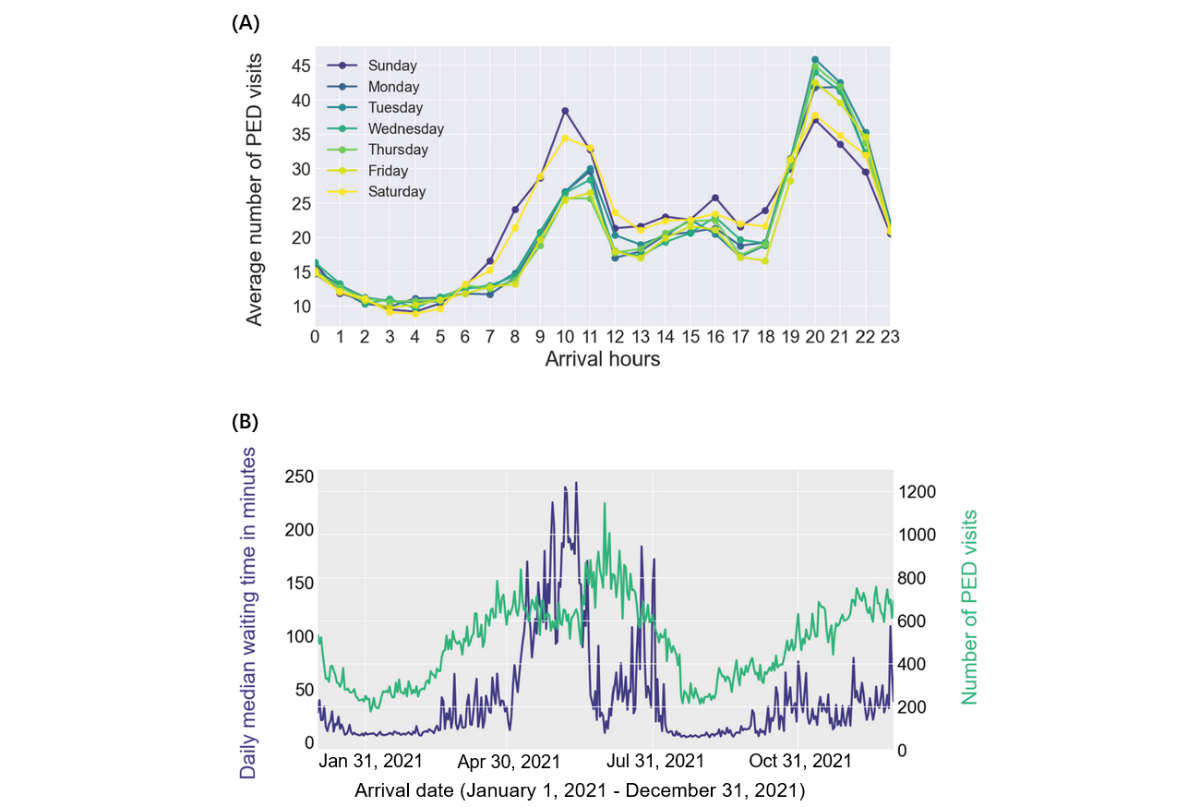
(A) Distribution of the temporal trends of the average number of PED visits for 24 hours of the day. The average number of arrivals in different hours of the day is represented by dotted lines with different colors. (B) The daily number of PED visits (green line) and the median waiting time (purple line) from January 1, 2021, to December 31, 2021. PED: pediatric emergency department.

On average, there were 490.54 PED visits/day on weekdays and 528.78 visits/day on weekends. Compared with weekdays, the number of PED visits was lower, with Sunday having the highest count of PED arrivals, averaging 532.02 visits/day (Figure 2B). Similarly, we observed longer waiting times on Sundays compared with other days (Figure 2C). Regarding the temporal trends of daily admissions, on average, the number of PED visits was high between 10:00 AM and 11:00 AM and between 8:00 PM and 9:00 PM, while it was low between 2:00 AM and 5:00 AM (Figure 3A). In summary, there was a consistent increase in PED arrivals starting at 6:00 AM, reaching a peak at 10:00 AM on weekends and 11:00 AM on weekdays. Afterward, the number of PED visits started to decrease. Several hours later, it began to rise again, reaching a second peak around 8:00 PM, marking the busiest period in the emergency room.

**Figure 4:**
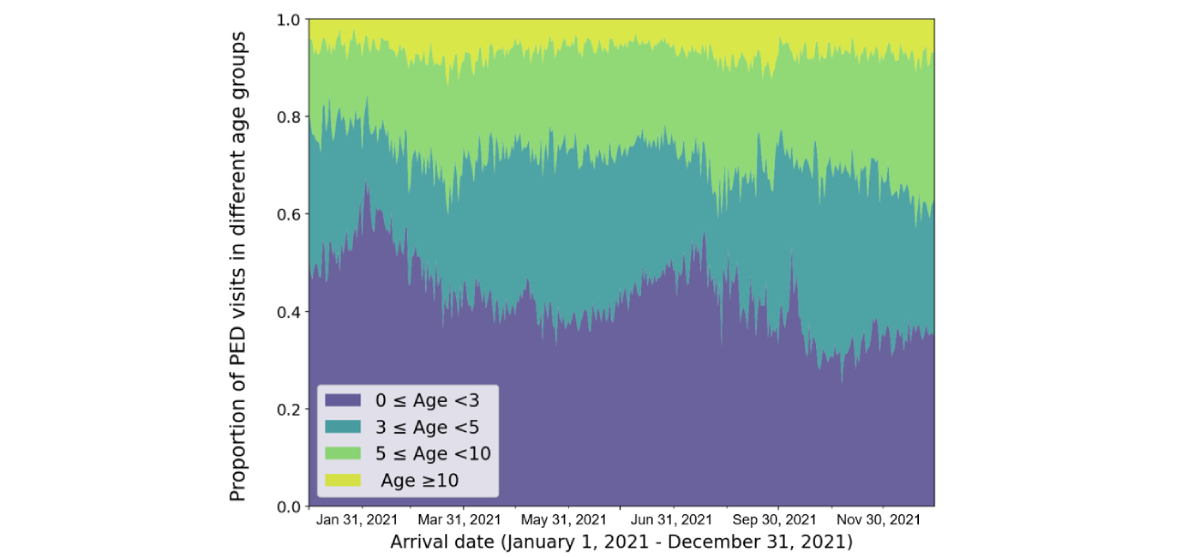
Temporal trends of the age distribution in pediatric emergency department visits from January 1, 2021, to December 31, 2021. The daily proportion of PED visits in different age groups is represented by different colors. PED: pediatric emergency department.

We also noted the influence of seasonality and holidays on PED arrivals. Throughout the year, the ED admissions were at their lowest during early February (coinciding with the Spring Festival and winter holidays) and late August (coinciding with the summer holidays; Figure 3B). The number of PED visits was generally higher during academic semesters compared with breaks. In particular, the daily number of PED visits increased steadily throughout March and April, peaking in early May and mid-June, respectively (Figure 3B). We observed a positive correlation between the number of PED visits and the average waiting time. For instance, the waiting time for patients admitted in May and June was higher than that in February, indicating longer waits during these 2 months. Similarly, as the number of PED visits decreased in late August, the median waiting time also decreased and remained relatively low around September. Subsequently, the daily number of PED visits gradually increased as temperatures dropped during the winter months. The seasonality of common pediatric infectious diseases may have contributed to these fluctuations in PED admissions. Additionally, the proportion of PED visits among those aged 3-5 years decreased from late January to February (Figure 4). During the same time interval, there was an increasing trend in the proportion of pediatric patients younger than 3 years, similar to what was observed in late August and early September. By contrast, the number of pediatric patients aged over 5 years remained relatively stable.

**Figure 5:**
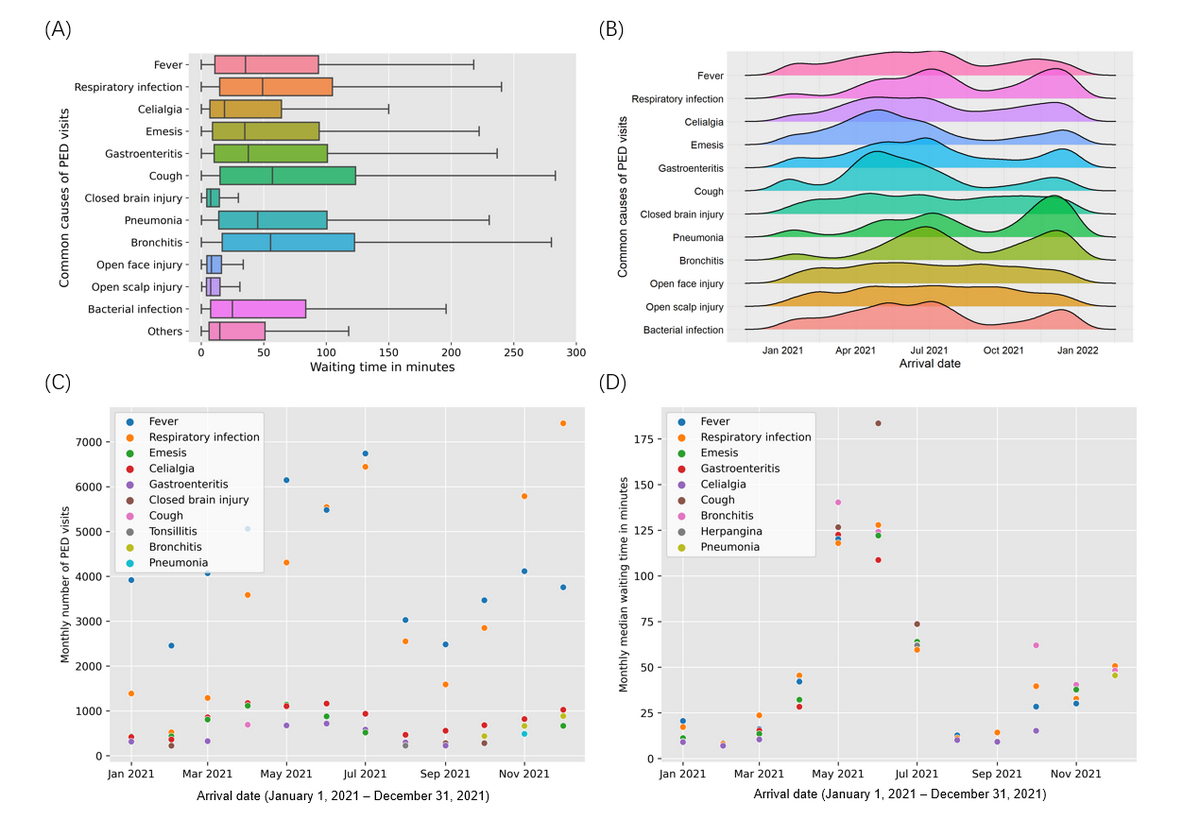
(A) Box plots for waiting time of PED patients with different diseases. (B). Ridgeline plots between arrival time and common diseases of PED visits. The density plot for each group is represented by different colors. (C) Scatter plot of the monthly top 5 common causes of PED visits and the corresponding number of PED visits. (D) Scatter plot of the top 5 causes with the highest monthly median waiting times, those top causes with monthly occurrences less than 300 were removed. PED: pediatric emergency department.

We also examined the temporal variation in common causes of pediatric emergency room visits and their associated waiting times. As depicted in Figure 5A, patients with open or closed injuries typically experience shorter waiting times compared with those with inflammations (eg, pneumonia and gastroenteritis), infections (eg, respiratory infection), or general symptoms (eg, cough and emesis). This finding aligns with the medium waiting times observed in different EDs, as shown in Table 1. Some common causes of PED visits exhibited seasonal epidemics. For instance, fever, respiratory infection, gastroenteritis, and bronchitis had peaks in April to July and October to December, mirroring the overall pattern of PED visit changes shown in Figure 3B. In summary, emesis and cough peaked from April to July, while pneumonia was more prevalent from October to December. On the contrary, closed brain injury, open face injury, and open scalp injury showed stable occurrence patterns throughout the year (Figure 5B). To explore the relationships between shifting trends in common causes of PED visits and extended waiting times, we identified the top 5 common causes for each month (Figure 5C), as well as the causes with the top 5 monthly median waiting times (Figure 5D). Figure 5C demonstrates that the occurrences of fever and respiratory infections fluctuated substantially over the year, whereas other causes remained relatively stable. Figure 5D indicates that causes with higher waiting times exhibited similar shifting trends over the entire year, which also aligns with the overall average waiting time changes shown in Figure 3B.

### Waiting Time Prediction and the Influential Factors

The performance metrics for waiting time prediction models are presented in Table 2. Overall, the LR, KNN, RF, and gradient boosting machine models demonstrated higher accuracy and goodness of fit compared with the commonly used rolling average method. This is because the rolling average estimator tends to underestimate or overestimate the waiting time more than other methods. Among the machine learning models, RF, LightGBM, and XGBoost outperformed LR, with no significant difference in performance, and their combined average performance increased *R*^2^ by approximately 29.33% (0.154/0.525) compared with LR and decreased RMSE by approximately 17.73% (8.951/50.481) in cross-validation. Machine learning models exhibited a broader range of values and had fewer predictions with high bias, as evidenced by the presence of more data points near the diagonal (refer to Multimedia Appendix 3). This aligns with existing studies demonstrating that machine learning models can capture more dynamic relationships between waiting time and related factors [22,25]. Among all the models, the XGBoost model achieved the highest *R*^2^ and the lowest RMSE, consistent with previous studies [25].

Furthermore, because the predictors were encouraged to provide precise predictions, especially for patients with longer waiting times, it is important to note that these patients, although they constitute a small proportion of overall visits, have a substantial impact on the long waiting queues in the PED. Detecting and addressing these outliers are crucial for improved PED admission management. We computed the mean absolute error scores of waiting time prediction models for the 3 subgroups with relatively higher waiting times within the overall distribution (50%-75%, waiting time 27.43-82.92 minutes; 75%-95%, waiting time 82.92-209.34 minutes; and 95%-100%, waiting time 209.34-479.97 minutes). The results in Table 3 demonstrate that both LR and machine learning models outperformed the traditional rolling average methods, particularly in the extremely high waiting time subgroups (75-95% and 95%-100%). Conversely, LightGBM and XGBoost consistently maintained acceptable prediction performance across all abnormal subgroups.

Subsequent feature importance analysis revealed that the number of triage green patients waiting to see a doctor was the most important factor, followed by the type of registered ED (refer to Multimedia Appendix 4).

**Figure 6:**
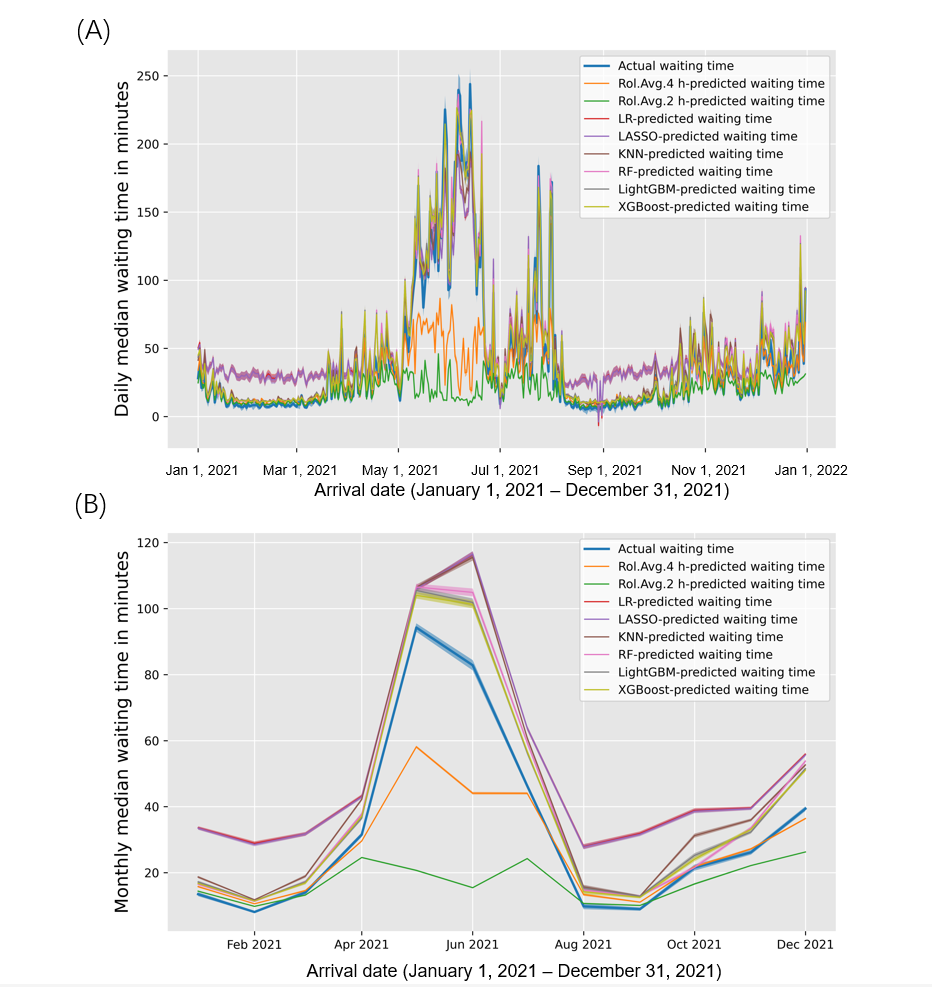
The distribution of the actual waiting time and the predicted waiting time by different models. (A) Daily median waiting times and their 95th quantile intervals for the actual waiting time and the predicted waiting time by different computational models are presented in the line chart with different colors. (B) Monthly median waiting times and their 95th quantile intervals for the actual waiting time and the predicted waiting time by different computational models are presented in the line chart with different colors. KNN: K-nearest neighbor; LASSO: least absolute shrinkage and selection operator; LightGBM: light gradient-boosting machine; LR: linear regression; RF: random forest; XGBoost: extreme gradient boosting.

We compared the distribution of predicted waiting times from different models with the actual observed data [29]. Daily and monthly median waiting times, along with their 95th percentile intervals, were calculated for both the observed data and predictions from various methods (Figure 6). In Figure 6A, at the daily level, most machine learning models (RF, LightGBM, and XGBoost) demonstrated similar distributions of predicted waiting times compared with actual waiting times. By contrast, the rolling average methods did not consistently predict observations during crowding periods (eg, from May to July), and LR methods performed poorly during relatively noncrowded periods (eg, from January to May and from August to November). Similar trends were observed at the monthly level (Figure 6B), with machine learning methods (RF, LightGBM, and XGBoost) exhibiting slightly larger prediction deviations in the peak months of PED admissions. This could be attributed to machine learning methods tending to provide higher waiting time predictions when there are a substantial number of data points with extremely high values, leading to larger distribution deviations from the ground truth.

**Table 2:** Performance of waiting time prediction models evaluated with cross-validation.^a^

Method type		*R*^2^ (SD)	Mean absolute error (SD)	Root-mean-square error (SD)
**Time series**				
	Rol.Avg. 4 h	0.066	41.381	70.819
	Rol.Avg. 2 h	–0.229	45.169	81.251
**Linear regression**				
	Linear regression	0.525 (<1×10^–4^)	33.734 (0.006)	50.481 (0.006)
	LASSO^b^	0.525 (<1×10^–4^)	33.656 (0.001)	50.480 (0.001)
**Machine learning**				
	K-nearest neighbor	0.472 (<1×10^–4^)	31.530 (0.007)	53.238 (0.015)
	Random forest	0.670 (<1×10^–4^)	23.337 (0.007)	42.111 (0.015)
	LightGBM^c^	0.682 (<1×10^–4^)	22.860 (0.010)	41.321 (0.012)
	XGBoost^d^	0.685 (<1×10^–4^)	22.587 (0.005)	41.157 (0.008)

^a^Both average performance and SD (in parenthesis) are reported only for the linear regression and machine learning methods.

^b^LASSO: least absolute shrinkage and selection operator.

^c^LightGBM: light gradient-boosting machine.

^d^XGBoost: extreme gradient boosting.

**Table 3:** The mean absolute error scores (SD) of different waiting time prediction models on 3 relatively higher waiting time subgroups (50%-75%, waiting time 27.43-82.92 minutes; 75%-95%, waiting time 82.92-209.34 minutes; and 95%-100%, waiting time 209.34-479.97 minutes).^a^

Method type		Subgroups		
		50%-75%	75%-95%	95%-100%
**Time series**				
	Rol.Avg. 4 h	24.425	75.889	231.180
	Rol.Avg. 2 h	26.046	101.230	268.496
**Linear regression**				
	Linear regression	20.875 (0.013)	34.781 (0.011)	116.596 (0.051)
	LASSO^b^	20.782 (0.001)	34.930 (0.001)	116.923 (0.003)
**Machine learning**				
	K-nearest neighbor	20.106 (0.015)	37.152 (0.025)	114.260 (0.090)
	Random forest	20.732 (0.006)	31.252 (0.021)	84.046 (0.029)
	LightGBM^c^	20.272 (0.012)	29.881 (0.028)	79.249 (0.086)
	XGBoost^d^	19.727 (0.006)	29.876 (0.015)	81.808 (0.063)

^a^The mean absolute errors and the SD (inside parentheses) are only reported for the linear regression and machine learning methods.

^b^LASSO: least absolute shrinkage and selection operator.

^c^LightGBM: light gradient-boosting machine.

^d^XGBoost: extreme gradient boosting.

## Discussion

### Principal Findings

As the number of pediatric emergency visits increases, the demand for cost-effective emergency medical care will also grow. The emergence of big data and the widespread adoption of electronic medical records have enabled us to address population health issues once considered insurmountable. Previous studies have shown that big data and machine learning are emerging trends and essential tools for modern health care systems, capable of creating models that perform at human-level accuracy [30-32]. Using clinical records from 183,024 PED visits, this retrospective study examined the characteristics and admission preferences of pediatric emergency visits while predicting their waiting times in EDs. Unlike previous studies that concentrated on patients with specific injuries in the ED, our study encompassed nearly all pediatric patients admitted to the PED. This approach has potential implications for the comprehensive management and resource allocation of pediatric emergency medical care [33-35].

Our results indicated a median waiting time from registration to the first physician visit of 27.53 minutes, which was lower than that reported in previous studies [36,37]. However, as the demand for emergency services increased, we observed prolonged waiting times during specific periods. For instance, the number of emergency room arrivals and the median waiting time both increased from February to June, with fluctuations. Furthermore, a similar phenomenon was observed as temperatures dropped in winter. As shown in previous studies [38,39], common causes of PED visits, such as fever and respiratory infection in this study, also exhibited seasonal patterns (Figure 5B), which could influence PED admissions. Additionally, our data revealed that the majority of PED visits were from children under the age of 5 years (130,423/183,024, 71.26%). As mentioned earlier, an increased proportion of PED visits in children younger than 3 years was observed from January to February, while those aged between 3 and 5 years showed a decreasing trend (Figure 4). This is mainly because children under the age of 3 years are typically cared for at home, and they are known to have a higher incidence of respiratory tract infections during the winter. The dry and cold conditions during winter are major factors contributing to increased respiratory tract infections as they enhance virus stability and transmission while weakening the host immune system. By contrast, kindergartens typically begin their winter break in mid-January, reducing the likelihood of cross-infections among children aged 3-5 years. A similar phenomenon was observed in the summer holidays. Given that children are particularly vulnerable to diseases influenced by climate and school holidays, several measures could enhance the management of pediatric emergency medicine and reduce PED visits. These include creating extensive administrative databases to monitor seasonal disease patterns [40,41], identifying peak months of PED admissions for proactive physician and nurse practitioner scheduling in emergency care, and disseminating infectious disease prevention and management reminders to parents and school caregivers through mobile health (mHealth) apps [42]. However, these strategies depend on extensive data integration and collaboration among various teams in the EDs.

We also examined the daily peak times for emergency rooms and inferred potential reasons for admission preferences. As shown in Figures 2B and C and 3A, emergency rooms are typically busier on weekends, particularly on Saturday and Sunday mornings. Patients may prefer the emergency room on weekends, possibly because it is less convenient to schedule appointments with primary care physicians on Saturdays or Sundays. The pediatric emergency room experiences its peak activity between 10:00 AM and 11:00 AM and 8:00 PM and 9:00 PM, with fewer admissions between 2:00 AM and 5:00 AM. Interestingly, on weekdays, the emergency room tends to become significantly more crowded in the evening, except for children with urgent illnesses. This increase in crowding could be attributed to the higher influx of nonemergency patients, some of whom may be seeking care to avoid rush hours. Our data indicated that 18.74% (34,339/183,204) of PED visits resulted in discharge without a prescription or further tests. In effect, most outpatient services were unavailable after 5 PM, and those who failed to attend outpatient registration were more likely to choose an emergency room instead. This also results in overcrowded pediatric emergency rooms, indicating that these emergency rooms have limited capacity to deliver timely and quality care. The analysis of peak ED hours can inform policy decisions regarding medical resource allocation and treatment process optimization to reduce patient waiting times. For instance, the PED can schedule additional nurses and proficient emergency physicians during daily peak periods to ensure effective emergency care for children. Furthermore, increasing outpatient capacity, such as offering evening outpatient services to assess nonemergency PED admissions, could divert patients from the ED, alleviating congestion and reducing waiting times for pediatric patients in need of urgent care. Given the necessity of hospitalization for some urgent patients after their initial management in the PED, streamlining the process for transitioning patients from the ED to the inpatient setting can enable ED providers and nurses to serve a larger volume of patients and enhance the efficiency of clinical care [43]. This strategy could free up more treatment rooms for ED arrivals but relies on collaboration between the PED and other inpatient units within the hospital. As part of medical process optimization, the PED can implement a streamlined admission process during daily peak hours by combining registration and triage simultaneously. Nursing staff should receive training to rapidly and accurately assess whether a patient has a genuine emergency, and dedicated pediatric staff should be assigned to provide appropriate medical care for urgent patients [44]. Additionally, it may be necessary to use information models to enhance the triage of seemingly stable patients, monitor their short-term clinical changes, and offer timely treatment [45]. Other promising solutions are implementing online triage systems for parents or guardians to assess the urgency of their child’s symptoms [46,47], offering telehealth services for emergency care [48], and deploying social robots to provide emotional support to children during overcrowding periods [49]. For instance, Rochat et al [50] introduced a patient-centered mHealth app that encompasses all aspects of pediatric emergency care. This app offers symptom recommendations, predicts potential waiting times, enables patients to temporarily wait outside after triage using a queue reminder system, and notifies the ED upon the patient’s arrival. However, this was only tested in a laboratory environment, and further implementation and assessment in large-scale real-world data are necessary.

In this study, we also noted that children with fewer PED visits had a higher percentage of longer wait times compared with children with frequent visits. While all pediatric patients arriving at the emergency room require immediate care, those with frequent PED readmissions may have a higher likelihood of needing urgent medical attention. Further studies are needed to assess and compare the causes and timing of readmissions. Additionally, we conducted waiting time prediction and investigated the factors influencing waiting times. The most important factor was the number of triage green PED visits in the waiting room, followed by the registered ED. Further queueing theory analysis could be applied to emergency wards to reduce patient waiting times [51]. Machine learning methods outperformed traditional regression models in predicting waiting times. Implementing these prediction models in clinical practice can assist emergency staff in accurately assessing waiting times and managing crowded patient flow more effectively. In future research, the integration of additional data types should be considered. For instance, prior studies have demonstrated that integrating historical daily ED arrivals and internet search data improved daily ED volume prediction [52], which could directly impact waiting times. Simultaneously, waiting time prediction can aid health care workers in pursuing quality improvement initiatives to reduce patient wait times. As mentioned earlier, the number of triage green PED visits in the waiting room emerged as the most influential factor in waiting times, indicating a certain extent of medical resource shortage. The hospital implemented some optimization strategies, such as increasing the number of emergency physicians on duty at the end of June. As depicted in Figure 3B, this led to a decrease in waiting times for PED visits despite an increase in the number of PED arrivals. However, the impact of these policies was limited. Because of the high volume of PED arrivals, even a small increase in the number of doctors resulted in small effects. The shortage of pediatricians in China is alarming, and training an adequate number of skilled pediatric physicians and nurses quickly is challenging [53,54]. Therefore, it is essential to consider useful strategies to address this situation. This may include implementing broader policies that offer better benefits and working conditions for childcare workers and encouraging more medical students to pursue careers in pediatrics, particularly in PEDs. Recently, several key medical schools in China have reintroduced full-time majors in clinical pediatric medicine and related subjects. This initiative aims to boost the supply of pediatric medical resources and services [53].

### Limitations

Our study has several limitations. First, this was a retrospective study based on patient admission data from a single hospital. As a result, our findings may be influenced by regional health care patterns and epidemiological trends specific to the hospital’s location. This may limit the generalizability of our results to other pediatric health care systems. Second, we relied on standard features commonly used in previous waiting time evaluation studies. While these modeling strategies have been validated, there may be room for further optimization in their practical application. Additionally, some potentially relevant information might have been overlooked. Lastly, it is advisable to collect more data to confirm the seasonality of common pediatric infectious diseases and assess the impact of the pandemic on PED admissions, disease patterns, and care-seeking behaviors. Previous studies, primarily based on data from 2020 or 2019, have demonstrated that COVID-19 led to a decrease in admissions and ED visits at children’s hospitals [55,56]. The implementation of nonpharmaceutical interventions during the pandemic also resulted in a reduction in admissions for respiratory diseases among children in China [57]. In our future study, we plan to collect records over a longer time span. This extended time frame will allow us to capture detailed temporal trends in emergency admissions and enhance the robustness of our waiting time prediction model. In future research, we will consider exploring additional machine learning methods, such as deep neural networks, for waiting time prediction. Deep learning techniques, which involve artificial neural networks, can automatically learn features from data and potentially improve prediction performance and generalization [58]. Despite the limitations, our study highlights current trends and issues within the Chinese pediatric population seeking emergency care. These findings suggest potential quality-of-care challenges in pediatric emergency medicine and may inform policy decisions.

### Conclusions

In conclusion, our real-world study analyzed the demographic characteristics, clinical presentations, and visit patterns of pediatric patients in the ED. We also developed machine learning models that effectively predict waiting times for doctor consultations. These models can aid physicians and patients in predicting busy periods and expected waiting times, facilitating timely decision-making for interventions. For children’s hospital EDs, they can be valuable in optimizing patient visit processes, significantly reducing waiting times, and enhancing overall pediatric emergency medical quality and service levels.

## Appendix

Multimedia Appendix 1 Description of the 27 predictors.

Multimedia Appendix 2 Details of the model hyper-parameter search experiments.

Multimedia Appendix 3 Predicted waiting time and true waiting time for different prediction models.

Multimedia Appendix 4 SHAP value from XGBoost shows the feature importance of the 27 different predictors.

## Abbreviations

ED: emergency department
ICD-10: International Classification of Diseases 10th Revision
KNN: K-nearest neighbor
LASSO: least absolute shrinkage and selection operator
LightGBM: light gradient-boosting machine
LR: linear regression
mHealth: mobile health
PED: pediatric emergency department
PEDOCS: Pediatric ED Overcrowding Score
RF: random forest
RMSE: root-mean-square error
XGBoost: extreme gradient boosting

## Availability of Data and Materials

The data that support the findings of this study are available in a deidentified form from the Children’s Hospital Capital Institute of Pediatrics, but restrictions apply to the availability of these data, which were used under license for this study, and so are not publicly available. Data are, however, available from the authors upon reasonable request and with permission of Children’s Hospital Capital Institute of Pediatrics, by sending a request to the corresponding author. The source codes of waiting time prediction models are released online [59].
