# Supplementary material for: Characteristics and Admission Preferences of Pediatric Emergency Patients and Their Waiting Time Prediction Using Electronic Medical Record Data: Retrospective Comparative Analysis
Source: J Med Internet Res. 2023 Nov 1;25:e49605. doi: 10.2196/49605 (PMC10652198; doi:10.2196/49605)
Supplement: Multimedia Appendix 1 [file jmir_v25i1e49605_app1.docx]

Description of the 27 predictors. Each predictor was calculated by the time when a current patient arrived at and was registered to the pediatric emergency department.

| Predictor | Description | Variable type |
| --- | --- | --- |
| age | age of the patient | Numerical |
| gender | gender of the patient | Categorical |
| times | PED visit frequency of the registered patient during the study period | Numerical |
| hour | hour of the day when the PED registration occurred | Numerical |
| month | month of the year when the PED registration occurred | Numerical |
| week | day of the week when the PED registration occurred | Numerical |
| holiday | a variable indicating whether the PED registration occurred on a public holiday | Categorical |
| triage | the following triage status of the patient | Categorical |
| department | the registered PED department | Categorical |
| doctor | number of doctors on duty (daytime/nighttime) | Numerical |
| arrivals.all | count for arrivals of the PED visits in the previous 1 hour | Numerical |
| arrivals.green | count for arrivals of triage green PED visits in the previous 1 hour | Numerical |
| arrivals.yellow | count for arrivals of triage yellow PED visits in the previous 1 hour | Numerical |
| left.green | count for the number of triage green PED visits who departed in the previous 1 hour | Numerical |
| left.yellow | count for the number of triage yellow PED visits who departed in the previous 1 hour | Numerical |
| left.wait.green | count for the number of triage green PED visits who experienced further consultation or treatment with a doctor, and departed in the previous 1 hour | Numerical |
| left.wait.yellow | count for the number of triage yellow PED visits who experienced further consultation or treatment with a doctor, and departed in the previous 1 hour | Numerical |
| left.nowait.green | count for the number of triage green PED visits who have not experience further consultation or treatment with a doctor, and departed in the previous 1 hour | Numerical |
| left.nowait.yellow | count for the number of triage yellow PED visits who have not experience further consultation or treatment with a doctor, and departed in the previous 1 hour | Numerical |
| treat.green | count for the number of triage green PED visits who have been seen by a doctor (consultant or treatment) and are still in the hospital. | Numerical |
| treat.yellow | count for the number of triage yellow PED visits who have been seen by a doctor (consultant or treatment) and are still in the hospital. | Numerical |
| wait.green | count for the number of triage green PED visits who have not yet been seen by a doctor (consultant or treatment) | Numerical |
| wait.yellow | count for the number of triage yellow PED visits who have not yet been seen by a doctor (consultant or treatment) | Numerical |
| 4h.green | count for the number of triage green PED visits who registered and have been seen by a doctor (consultant or treatment) in the previous 4 hours | Numerical |
| 4h.yellow | count for the number of triage yellow PED visits who registered and have been seen by a doctor (consultant or treatment) in the previous 4 hours | Numerical |
| t.avg4h.green | the average waiting time for the triage green PED visits who registered and have been seen by a doctor (consultant or treatment) in the previous 4 hours | Numerical |
| t.avg4h.yellow | the average waiting time for the triage yellow PED visits who registered and have been seen by a doctor (consultant or treatment) in the previous 4 hours | Numerical |
| Total number of predictors | | 27 |
