# Supplementary material for: Characteristics and Admission Preferences of Pediatric Emergency Patients and Their Waiting Time Prediction Using Electronic Medical Record Data: Retrospective Comparative Analysis
Source: J Med Internet Res. 2023 Nov 1;25:e49605. doi: 10.2196/49605 (PMC10652198; doi:10.2196/49605)
Supplement: Multimedia Appendix 2 [file jmir_v25i1e49605_app2.docx]

Details of the model hyper-parameter search experiments.

| Model | Hyperparameters |
| --- | --- |
| Rol.Avg.4h | None |
| Rol.Avg.2h | None |
| LR | normalize: True |
| LASSO | normalize: True |
| KNN | n_estimators: 2-20(step=1),  leaf_size: 2-20(step=2) |
| RF | n_estimators: 100-300(step=50),  max_depth: 1-10(step=1) |
| LightGBM | n_estimators: 250  max_depth: 3-8(step=1),  num_leaves: 5-100(step=5),  feature_fraction: 0.6-1.0(step=0.1),  bagging_fraction: 0.6-1.0(step=0.1),  bagging_freq: 0-81(step=20),  learning_rate: [0.001, 0.003, 0.005, 0.008, 0.01, 0.03, 0.05, 0.08, 0.1] |
| XGBoost | n_estimators: [80, 100, 150, 200],  max_depth: 3-10(step=2),  min_child_weight: 1-6(step=2),  learning_rate: [0.001, 0.003, 0.005, 0.008, 0.01, 0.03, 0.05, 0.08, 0.1] |
