# Supplementary material for: Characteristics and Admission Preferences of Pediatric Emergency Patients and Their Waiting Time Prediction Using Electronic Medical Record Data: Retrospective Comparative Analysis
Source: J Med Internet Res. 2023 Nov 1;25:e49605. doi: 10.2196/49605 (PMC10652198; doi:10.2196/49605)

Predicted waiting time and true waiting time for different prediction models. Each dot represents an observation in the PED visits data set. Triage categories of the PED visits are represented by different colors (green and yellow). The predicted value for each observation was picked from one of the repeated cross-validations. The diagonal line in each plot represents the line where predicted waiting time equals to actual waiting time.


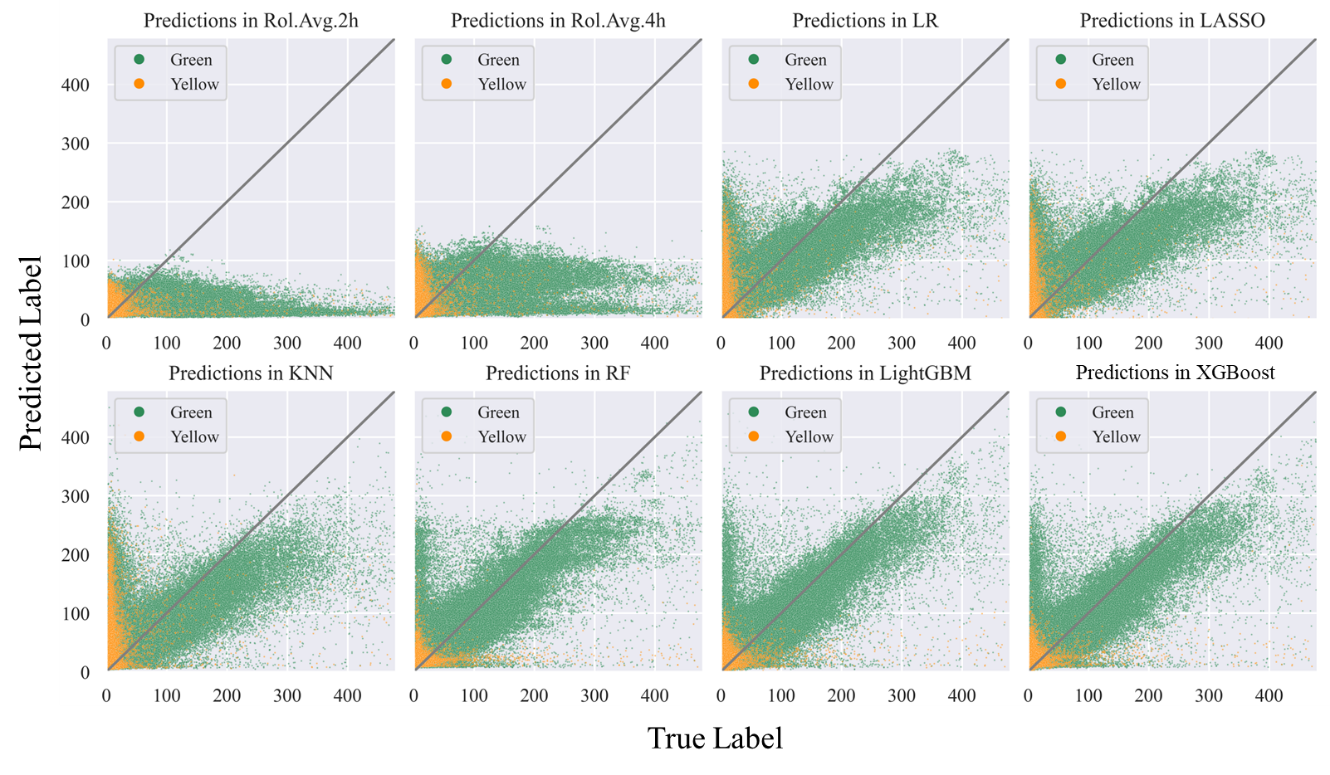

Supplement: Multimedia Appendix 3 [file jmir_v25i1e49605_app3.docx]
