# Supplementary figures and images for: Characteristics and Admission Preferences of Pediatric Emergency Patients and Their Waiting Time Prediction Using Electronic Medical Record Data: Retrospective Comparative Analysis
Source: J Med Internet Res. 2023 Nov 1;25:e49605. doi: 10.2196/49605 (PMC10652198; doi:10.2196/49605)

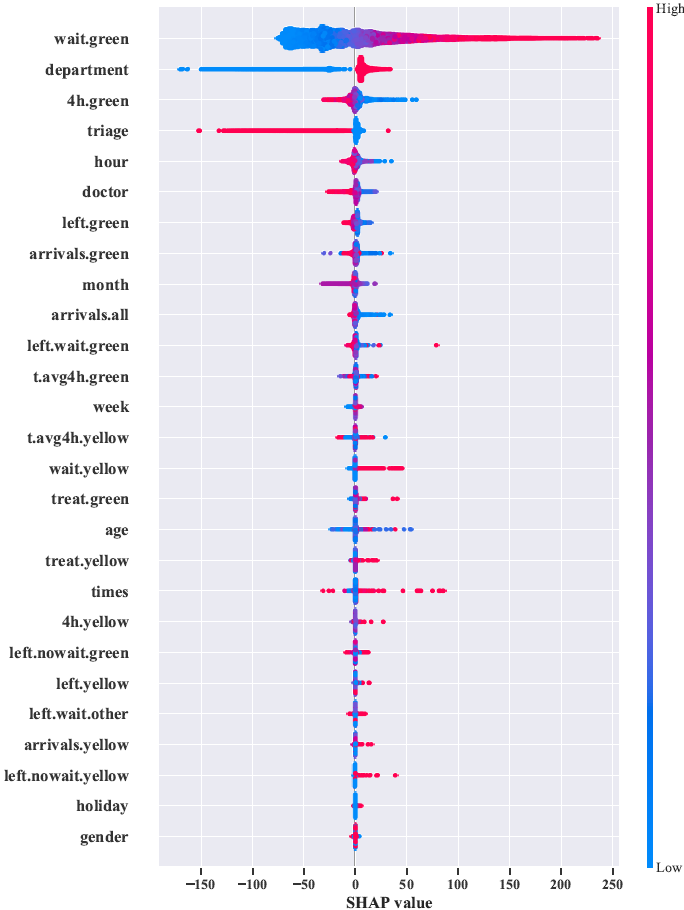
SHAP value from XGBoost shows the feature importance of the 27 different predictors.

Supplement: Multimedia Appendix 4 [file jmir_v25i1e49605_app4.docx]
